# A deep learning solution for crystallographic structure determination

**DOI:** 10.1107/S2052252523004293

**Published:** 2023-07-01

**Authors:** Tom Pan, Shikai Jin, Mitchell D. Miller, Anastasios Kyrillidis, George N. Phillips

**Affiliations:** a Department of Computer Science, Rice University, Houston, Texas, USA; b Department of Biosciences, Rice University, Houston, Texas, USA; c Center for Theoretical Biological Physics, Rice University, Houston, Texas, USA; d Department of Chemistry, Rice University, Houston, Texas, USA; University of Chicago, USA

**Keywords:** structure prediction, structure determination, X-ray crystallography, deep learning

## Abstract

The general *de novo* solution of the crystallographic phase problem is difficult and only possible under certain conditions. This paper develops an initial pathway to a deep learning neural network approach for the phase problem in protein crystallography, based on a synthetic dataset of small fragments derived from a large well curated subset of solved structures in the Protein Data Bank (PDB). In particular, electron-density estimates of simple artificial systems are produced directly from corresponding Patterson maps using a convolutional neural network architecture as a proof of concept.

## Abstract 2

Machine learning methods have been applied to solve simple Patterson maps. The results demonstrate the potential to use neural networks for solving the phase problem in crystallography.

## 1. Introduction

Proteins are an important class of organic macromolecules in living systems as they are the driving force behind the vast majority of cellular processes. Determining the structure of a protein is one of the classic problems in biology, as a protein’s function is specified by its structure. In essence, proteins are polymers of relatively small organic molecules called amino acids, of which there are 20 that are considered to be in the standard set. However, these underlying polypeptide chains always fold into complex three-dimensional structures (as well as potential complexes) to form their active conformations; for example see Petsko & Ringe (2008 ▸). Thus, biologists would like to have a standard method for experimentally determining and viewing a protein’s overall structure.

### 1.1. Crystallographic phase problem

X-ray crystallography has been the most commonly used method to determine protein structure for over 60 years (Berman *et al.*, 2004 ▸). In review, an X-ray crystallography experiment measures a diffraction pattern which consists of a set of spots, *e.g.* on a detector surface. Each spot (known as a reflection) is denoted by three indices *h*, *k*, *l*, known as Miller indices. These correspond to sets of parallel planes within the protein crystal’s unit cell that contributed to producing the reflections, and the set of possible *h*, *k*, *l* values is determined by the radial extent of the observed diffraction pattern. Any reflection has an underlying mathematical representation, known as a structure factor, dependent on the locations and scattering factors of all the atoms within the crystal’s unit cell,

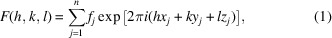

where the scattering factor and location of atom *j* are *f*_*j*_ and (*x*_*j*_, *y*_*j*_, *z*_*j*_), respectively. A structure factor *F*(*h*, *k*, *l*) has both an amplitude and a phase component (denoted by ϕ) and thus can be considered a complex number. Furthermore, suppose we knew both components of the structure factors corresponding to all of the reflections within a crystal’s diffraction pattern. Then, in order to produce an accurate estimate of the electron density ρ at any point (*x*, *y*, *z*) within the crystal’s unit cell, we would only need to take an inverse Fourier transform of all of these structure factors, 

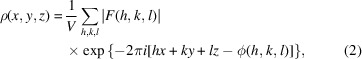

where *V* is the volume of the unit cell. The amplitude |*F*(*h*, *k*, *l*)| of any structure factor is easy to determine, as it is simply proportional to the square root of the measured intensity of the corresponding reflection. Unfortunately, it is impossible to determine directly the phase ϕ(*h*, *k*, *l*) of a structure factor, and this is what is well known as the crystallographic phase problem; see for example Lattman & Loll (2008 ▸).

A few methods of varying popularity have been developed for solving the crystallographic phase problem for determining protein structures. The three most commonly used methods are isomorphous replacement, anomalous scattering and molecular replacement (Lattman & Loll, 2008 ▸; Jin *et al.*, 2020 ▸). Also, for small molecules that diffract to true atomic resolution, direct methods of solving the phase problem are available (Karle & Hauptman, 1950 ▸). However, this method does not work for protein crystallography except in the rarest of cases, as the requirement that atoms be completely resolved as separate objects is rarely attainable. If this does not hold, then the probabilistic principle on which these methods are dependent (Sayre, 1952 ▸) also does not hold.

There have also been several methods developed that attempt to solve the problem of determining phase information given direct access only to intensity measurements in a more general setting (Fienup, 1982 ▸), better known as phase retrieval in non-crystallographic contexts. However, none have seen widespread use in the X-ray crystallography context as they either assume a more continuous sampling of intensities than possible in our setting, or were developed for use in specific non-crystallographic fields of physics (Guo *et al.*, 2021 ▸; Kappeler *et al.*, 2017 ▸; Rivenson *et al.*, 2018 ▸). The most well known of these is probably the iterative non-convex Gerchberg–Saxton (G-S) (Zalevsky *et al.*, 1996 ▸; Fienup, 1982 ▸) algorithm, which has been applied in various optical settings such as electron microscopy. However, it has not been applied to crystallography since it requires more input measurements than would be available. Fienup (1982 ▸) also extended the G-S algorithm to work better in settings similar to X-ray crystallography. Similar methods to that developed by Fienup have occasionally been applied to solve the crystallographic phase problem, but only in special cases where the crystals have very high solvent content (He & Su, 2015 ▸; He *et al.*, 2016*a* ▸; Kingston & Millane, 2022 ▸).

### 1.2. Patterson methods

Many of the commonly used procedures to solve the phase problem of X-ray crystallography make use of another mathematical representation called a Patterson function (Patterson, 1934 ▸). It has long been known as a useful tool for crystallographers, although traditionally it has not been used to solve the crystallographic phase problem directly for large molecules such as proteins. Our project aims to provide a method of solving structures via direct interpretation of Patterson function applications.

Essentially, the Patterson function is a simplified variation of the Fourier transform from structure factors to electron density, in which all structure factor amplitudes are squared and all phases are set to zero (*i.e.* ignored), 

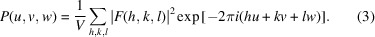

Locations in the Patterson unit cell are usually denoted by indices *u*, *v*, *w* to distinguish them from locations in the true unit cell, as the two have the exact same dimensions. A simplification using Euler’s formula then gives the most common form of the Patterson function, 

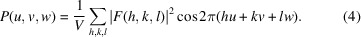




Applying the Patterson function over all unit-cell locations *u*, *v*, *w* creates what is known as a Patterson map, which is periodic with the same dimensions as the crystal’s unit cell. The Patterson function can be computed without direct access to any phase information of the structure factors. Thus, a Patterson map can be obtained directly from diffraction data without the need for additional experiments or outside information. These Patterson maps, which are formally autocorrelations of the corresponding electron densities, can be considered three-dimensional images that capture indirect information about the structure within the corresponding protein crystal’s unit cell.

Therefore, Patterson maps are natural inputs into a well known class of machine learning models. If we can provide Patterson maps as inputs into such a machine learning framework and obtain suitably accurate electron-density map predictions, then we can bypass the crystallographic phase problem and potentially save time and effort.

**Figure 1:**
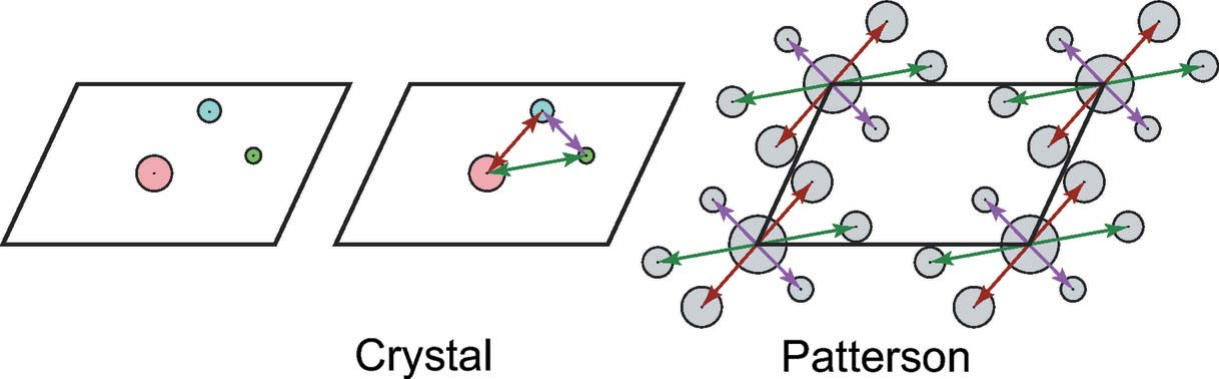
An example of the corresponding Patterson map (right) given atomic locations (left) and the interatomic vectors formed by them (middle).

It can be shown that a Patterson map will have peaks (which are also called Patterson vectors) at positions corresponding to interatomic vectors within the original crystal’s unit cell (Lattman & Loll, 2008 ▸), as shown in Fig. 1 ▸. Furthermore, the height of any such peak is proportional to the product of the atomic numbers of the two atoms in the corresponding atomic pair. This means that if a protein crystal contains *n* atoms in its unit cell, the resulting Patterson map will contain order *n* ^2^ peaks within its unit cell. This, along with substantial peak overlap, means that Patterson maps for large organic molecules such as proteins are considered to be uninterpretable for humans. Furthermore, given the nature of Patterson vectors, Patterson maps are invariant to translation of the entire contents of the original crystal’s unit cell.

### 1.3. Machine learning formulation

We want to generate predictions of the values at all locations (*x*, *y*, *z*) of an electron-density map given the values at all locations (*u*, *v*, *w*) of the corresponding Patterson map. Due to the complexity of such a transformation, we would not want to solve some optimization problem to determine all of its aspects explicitly ourselves. Instead, we want to automate its specification by making use of supervised parametric machine learning. This technique, based on the statistical principle of empirical risk minimization, involves a computer system automatically optimizing (‘learning’) the parameters 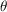 (often called weights) of a transformation 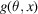 , where *x* denotes an input into the transformation (Goodfellow *et al.*, 2016 ▸). In our case, this is a Patterson map. This optimization is done in an iterative procedure where the predictions given the current parameter values are compared with the true values via a loss function 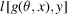 , where *y* denotes the desired output corresponding to input *x*. In our case, this is the corresponding true electron-density map. Formally, given a set of *n* input/output examples 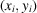 , the aim is to find parameter values 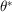 such that

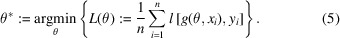

The parameter values can be updated by an optimization algorithm, such as the classic stochastic gradient descent (Robbins, 1951 ▸). This entire process is called training.

In particular, we are making use of what is now the most commonly used machine learning architecture – that of the neural network. Neural networks allow us to express a complex overall transformation as a composition of simpler, often standardized, transformations. These constituent functions are known as layers, and the output of one layer is passed through a nonlinear activation function before being given to the next one. In the simplest and earliest developed layer, the fully connected (FC) one, any output of the layer depends on all of the layer inputs (Goodfellow *et al.*, 2016 ▸). However, this results in very slow training for large neural networks.

Since the inputs we work with have a 3D shape and their elements have spatial meaning, we can instead make use of 3D convolutional layers as our default layers. Such layers enforce both sparse connectivity and weight sharing. A location in the output of a convolutional layer only depends on a relatively small spatially localized subset of the input locations. Also, the weights that these input values are multiplied by are shared across all output locations (Goodfellow *et al.*, 2016 ▸). Recently, several convolutional deep neural network approaches have been developed for phase retrieval within various fields of optics in order to bypass the demanding computational requirements of modern convex programming methods. One project (Kappeler *et al.*, 2017 ▸) used a very simple convolutional neural network with only three convolutional layers to perform phase retrieval in the Fourier ptychography setting. Meanwhile, another report (Rivenson *et al.*, 2018 ▸) used a more complex model architecture, consisting of several convolutional networks with residual blocks in parallel, to reconstruct holographic images from corresponding hologram intensities. In both the Kappeler and Rivenson models, multiple similar input intensities are given to the machine learning model, unlike our approach which is to provide a single input Patterson map.

The only previous work directly related to our particular line of inquiry of applying machine learning to solve the phase problem of X-ray crystallography using Patterson maps was done by David Hurwitz, who used a simple 3D convolutional model to predict the locations of randomly positioned sets of ‘atoms’ within a 3D space given the corresponding Patterson maps (Hurwitz, 2020 ▸). He determined several potential issues that could arise from the inherent properties of Patterson maps, which then lead to ambiguity in their interpretation. We have either addressed these issues or found that we could ignore them to a certain extent.

In this project, we have devised a deep learning approach for the direct interpretation of simple Patterson maps. We developed a standardized procedure for generating datasets with examples consisting of calculated electron densities of short adjacent peptides and their corresponding Patterson maps, derived from existing Protein Data Bank (PDB; Berman *et al.*, 2000 ▸) entries. We trained a convolutional neural network model on several such datasets, where the inputs to our model are Patterson maps and the predictions are electron densities, and have obtained successful results on a few initial datasets. We found that several difficulties arising during training on Patterson maps of randomly placed atoms can be alleviated due to the innate structural properties of natural amino acid residues. Overall, we have designed a new deep learning approach to bypass the phase problem and have achieved a successful solution on simple dipeptide examples.

## 2. Methods

### 2.1. Choice of model architecture

Because of the shape of our Patterson map inputs and electron-density outputs, we use the well known convolutional neural network model architecture. Such models are most commonly used for image recognition and classification purposes. Thereby, they usually contain some FC layers at the very end of the model (Wang *et al.*, 2020 ▸) and are often referred to as encoders. But we do not want our model to produce just one or a vector of values for a given input. Instead, we want to produce outputs of the same dimensionality as our inputs. Therefore, a natural choice of model architecture is the U-net, which was first introduced for a biosciences application (Ronneberger *et al.*, 2015 ▸) and is an example of an encoder–decoder network. In particular, almost all layers of our model are convolutional, except for those that perform downsampling and upsampling operations.

**Figure 2:**
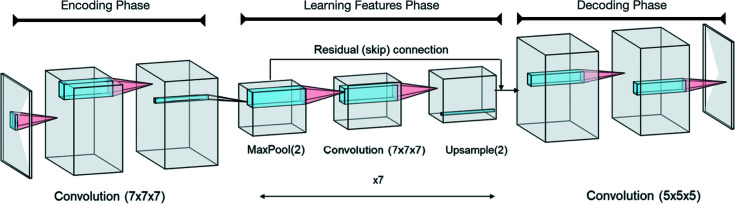
The neural network architecture of our proposal, showing the height, width and channel dimensions. The depth dimension is not shown. Generated using the *NN-SVG* online tool (LeNail, 2019 ▸).

Thus, our current model architecture is an extension of the architecture proposed by Hurwitz. Although it is a 3D convolutional U-net as well, we also make use of residual connections (He *et al.*, 2016*b* ▸) which have seen widespread use in convolutional neural networks. It is divided into three phases and is implemented in the *PyTorch* machine learning framework (Paszke *et al.*, 2019 ▸) for the Python programming language. A representation of the model, in which the depth dimension of the Patterson and electron-density maps is not displayed, is shown in Fig. 2 ▸.

### 2.2. Detailed description of current model architecture

The phases of our model are the Encoding, Learning Features and Decoding phases. The Encoding phase consists of two 7×7×7 convolutional layers, both followed by batch normalization and a ReLU activation. Afterwards, a max pooling operation with kernel size 2×2×2 and stride 2 is used to reduce the height, width and depth dimensions by a factor of 2. The Learning Features phase consists of a sequence of several residual blocks. Each of these blocks consists of a 7×7×7 convolutional layer with batch normalization and ReLU activation, followed by another 7×7×7 convolutional layer with batch normalization but no activation. [In later versions of our model, we introduced a squeeze and excitation block (Hu *et al.*, 2018 ▸) at this point, applied with the channel dimension reduced by a factor of 2. This is a method to reweight each channel based on the global information present in the channel.] The residual skip connection is then applied, followed by a ReLU activation. At the end of this phase, a naive upsampling operation is used to increase the height, width and depth dimensions by a factor of 2, restoring the original dimensions. The Decoding phase consists of two 5×5×5 convolutional layers. The first is followed by batch normalization and a ReLU activation, while the second produces the model predictions. Since all elements of the target outputs were constrained to be in the range [−1, 1], we apply a final tanh activation function after this layer. There are about three million trainable parameters in our original convolutional U-net model. See the supporting information for more details on the model architecture.

In all convolutional layers, the input is ‘same’ padded to preserve dimensionality. The convolutional layers in the Encoding and Learning Features phases are padded using *PyTorch*’s circular padding scheme to account for the periodic nature of the input Patterson maps. Furthermore, all convolutional layers were initialized using the kaiming_normal function of the default torch.nn module, which uses the He initialization scheme with a normal distribution (He *et al.*, 2015 ▸). Also, all convolutional layers except the last have multiple output channels. Currently, all inputs and outputs to the convolutional neural network are assumed to be of a constant cubic size. The loss function used to compare our model’s output predictions with the true electron-density maps was the mean-squared error function. Given an input Patterson map *p*, a corresponding electron-density map *e* and current model parameters 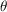 ,

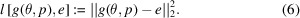




### 2.3. Datasets and data generation process

We generated several synthetic datasets that we used to train and test our machine learning model. All of the input and output maps we generated for our datasets were derived from actual PDB entries of proteins solved by X-ray crystallography (Berman *et al.*, 2004 ▸). A total of ∼24 000 such protein structures were curated, based on criteria such as sequence length, to form the basis for the examples of all our datasets. For each of these, non-protein atoms in the PDB file were removed, and then dipeptides of adjacent amino acid residues were randomly extracted to a new file with a fixed unit cell. Since one issue leading to potential ambiguity in interpreting Patterson maps is their invariance to translation of the corresponding electron density (Hurwitz, 2020 ▸), we decided to center each such dipeptide according to its center of mass in its unit cell. Although this meant that our model’s predicted electron densities would always be roughly centered in the unit cell, we did not consider this to be a particular issue with respect to realism. Structure factors for each of the dipeptide examples were then generated to 1.5 Å resolution, and electron-density and Patterson maps for each example were obtained from those structure factors. These maps were then converted into *PyTorch* tensors. Finally, we normalized the values in each of the tensors to be in the range [−1, 1] after determining the maximum and minimum values present in each. Additional details of our data generation process can be found in the supporting information.

Another issue brought up by Hurwitz regarding ambiguity in Patterson map interpretation is the fact that an electron density will always have the exact same Patterson map as its corresponding centrosymmetry-related electron density (Hurwitz, 2020 ▸). One method to address this ambiguity is always to combine a set of atoms with the set of its centrosymmetry-related atoms into a single example output. However, this workaround requires additional post-processing to separate the original and centrosymmetric densities for each of the model’s predictions. But here we are working with molecular structures rather than randomly placed data, so we can exploit certain known properties. In particular, we know that all proteinogenic amino acids are found in only one possible enantiomeric configuration (Helmenstine, 2021 ▸). Although the mirror-image symmetry of enantiomers is not exactly the same as centrosymmetry, we still hypothesized that the fixed chirality of amino acids was close enough to cause our model to learn a standard stereochemistry, thus allowing us to use individual dipeptide electron densities instead of applying Hurwitz’s workaround.

### 2.4. Training and analysis

We used the Pearson correlation coefficient as an additional metric to compare our model’s predictions with the corresponding desired electron densities during training. This metric involves all of the relevant elements *x*_*i*_ and *y*_*i*_ of the predicted and actual electron-density map tensors, respectively, as well as their average values 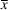 and 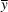 : 

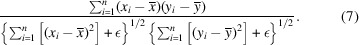

We also performed phase error analysis for our model’s post-training predictions using the *cphasematch* program of the *CCP*4 program suite (Cowtan, 2011 ▸; Agirre *et al.*, 2023 ▸). We performed all our training runs on a single NVIDIA GeForce GTX Titan GPU, making use of *PyTorch*’s CUDA library (NVIDIA *et al.*, 2020 ▸).

## 3. Results

### 3.1. Dialanine experiments

As in previous work (Hurwitz, 2020 ▸), we have implemented cases using synthetic training and test sets for the successful interpretation of Patterson functions using a convolutional neural network (CNN). As stated above, these are generated from a few thousand instances of dipeptide configurations taken from a randomly selected set of PDB entries. For our first dataset, referred to here as Dataset 1a, we converted the extracted dipeptides to dialanine by truncation at the C_β_ atom and renaming. This was done to simplify the initial problem, as alanine is among the smallest and simplest proteinogenic amino acids. To simplify the problem further, we placed all of the dialanines in a *P*1 unit cell with cubic dimensions. We also considered Hurwitz’s suggestion for eliminating yet another source of ambiguity in Patterson map interpretation – the fact that, since a Patterson map is periodic and its peaks correspond to vectors, it can be ambiguous from which corner of the Patterson map a Patterson vector originates (Hurwitz, 2020 ▸). Thus, for this initial dataset, we artificially enlarged the unit cells of our dialanine examples with enough empty space on all sides so that any Patterson vector in the resulting Patterson maps could only originate from the corner that it is closest to.

A total of 28 470 training and 3147 validation/test examples of unit-cell size 20 × 20 × 20 Å were generated. As already stated, the loss function was originally the simple mean-square error between the predicted maps and the original electron-density maps. We also calculated the average Pearson correlation coefficient between the central 6 × 6 × 6 Å regions of the learned and original electron-density maps over the set of validation examples after every training epoch, as the remaining portions of the maps were empty.

**Figure 3:**
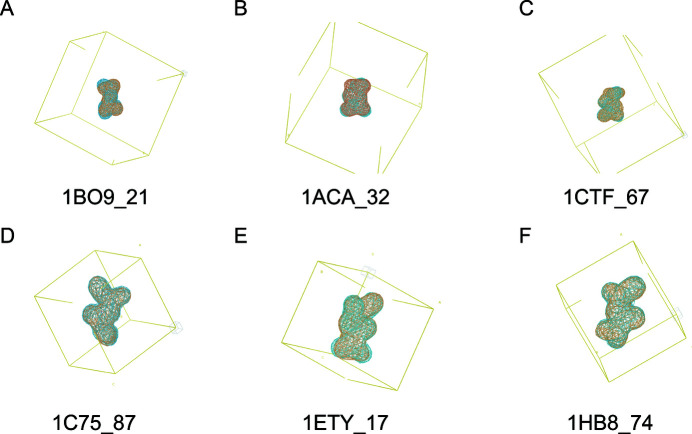
Examples of predictions on dialanine Datasets 1a (first row) and 1b (second row). The desired electron density is shown in blue and the predicted electron density is shown in orange. The labels indicate the PDB IDs and the starting residue index of the electron-density map.

Following tests using a learning rate finder tool, we settled on a final learning rate schedule of a 0.86 exponential decay for the first 12 training epochs, followed by a 0.9991 exponential decay for the remaining epochs. After training for 1000 epochs with a batch size of 146 (effectively 438 due to gradient accumulation) using the *Adam* optimizer (Kingma & Ba, 2015 ▸), predictions on the test set were created using the learned weights. The CNN was able to produce correct solutions, as demonstrated by comparison of the predictions with the corresponding known electron densities (Fig. 3 ▸). The median correlation coefficient for these test set predictions relative to the corresponding known dialanine densities after training was over 0.9, indicating success. This also more or less confirmed our hypothesis about centrosymmetry-induced ambiguity in Patterson map interpretation.

However, actual crystallographic protein structures are not surrounded by empty space, so we knew that continuing to eliminate ambiguity completely in the Patterson vector origin by surrounding our dipeptides with significant amounts of empty space would not be a viable option if we wanted our model to work on real-world data. Since organic molecules are structured rather than consisting of randomly placed atoms, we predicted that our model could handle some ambiguity in the Patterson vector origin after greatly reducing the amount of empty space we introduced. This hypothesis was shown to be correct by the high correlations in all the tests we performed. In fact, reducing the cell size and thus making the origin definition harder actually helped the training efficacy for Dataset 1b, although for 1b the training set size was also increased, which is also likely to have contributed to the improved performance (see Table 1 ▸).

**Figure 4:**
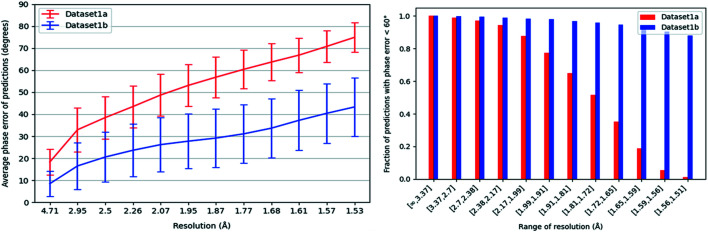
(Left) A plot of the average phase error of validation set predictions on Datasets 1a and 1b (dialanine datasets) against resolution. For the predictions on Dataset 1b, the average phase error remains below 60° for the entire range of resolution. (Right) The fraction of validation set predictions for which the phase error is <60° at various ranges of resolution, for Dataset 1a and 1b predictions.

Starting with Dataset 1b, we calculated correlation coefficients using the entire boxes rather than only the central regions, as accounting for a significant amount of surrounding empty space was no longer necessary. For both Datasets 1a and 1b, we calculated the average phase error over all predictions on validation set examples at various resolutions and created the plot shown on the left in Fig. 4 ▸. There are clearly better phase error results on the predictions for Dataset 1b. Since the average phase errors remain low even at high resolution, we conclude that our model’s predictions on Dataset 1b match even the finer details of the corresponding actual electron densities in general. This is not surprising given the simple structure of alanine residues. For both datasets, we also created a plot of the fraction of validation set predictions for which the phase error is <60° at various ranges of resolution, as shown on the right in Fig. 4 ▸. For Dataset 1a, we see a gradual decrease in this fraction at higher bins of resolution. However, for Dataset 1b we still have a very high fraction of predictions with phase error <60°, even at the highest ranges of resolution. Also, for both datasets the fraction of predictions with low phase error is very high at the lowest bins of resolution. Overall, this shows that the model is able to reproduce the general shape of the desired electron densities on both datasets, but is able to produce higher-resolution predictions (*i.e.* it more accurately generates finer details) after training on Dataset 1b.

### 3.2. Dipeptide experiment

After our initial success on the dialanine datasets, we switched to Dataset 2, consisting of dipeptide examples where each dipeptide could be any of the 20 standard proteinogenic amino acids rather than just alanine. The examples we produced for Dataset 2 were slightly larger than for the dialanine datasets, at 12 × 12 × 12 Å, to limit the number of dipeptides containing larger amino acids that would be rejected due to spatial clashes with neighboring unit cells. As a result of this greatly increased variability in our examples, this is considered to be a much more difficult problem. With the same model as we used in the dialanine experiments, we found that the validation set metrics plateaued after relatively few training epochs. Thus, we decided to increase our model complexity and thereby address the increased problem complexity.

The improvement was done by increasing the number of channels in our convolutional layers. Layers with 23 original channels were increased to 25 channels and layers with 25 original channels were increased to 30. We increased the number of residual blocks in the Learning Features phase from seven to eight. We also introduced squeeze and excitation blocks into the residual blocks and switched to the *AdamW* optimizer (Loshchilov & Hutter, 2019 ▸) with a weight decay parameter of 3 × 10^−2^. Furthermore, we began augmenting the loss function by adding 1 minus the calculated Pearson correlation coefficient to the MSE loss for each training example to produce a weighted combined loss function (with the weight heavily in favor of the MSE loss). We also experimented with introducing *Inception* v1 modules (Szegedy *et al.*, 2015 ▸) in place of simple convolutional layers in our residual blocks, but found slightly worse performance than without. This suggests that, given our current problem, the 7×7×7 kernels we use in the Learning Features phase are the optimal size.

**Figure 5:**
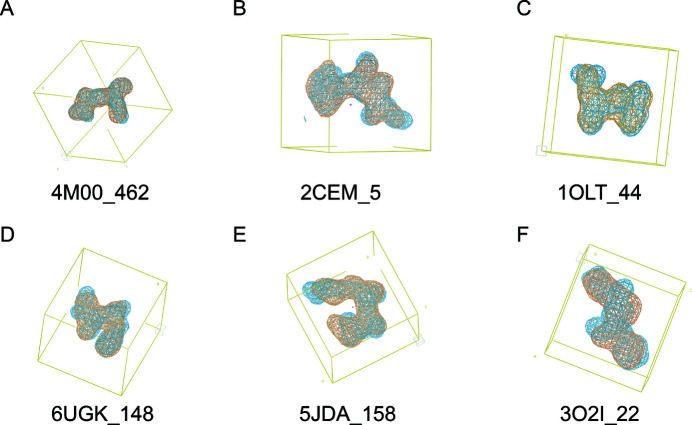
Examples of predictions on dipeptide Dataset 2. The desired electron density is shown in blue and the predicted electron density is shown in orange. The labels indicate the PDB IDs and the starting residue index of the electron-density map.

We also further increased the size of both the training and validation sets for this dataset, which ended up with 424 096 training and 47 126 test examples. After training for 200 epochs with a batch size of 58 (effectively 928), we obtained a median test set Pearson correlation coefficient of about 0.87, also indicating success. We also slightly modified the learning rate schedule, which now has a 0.91 exponential decay for the first 18 epochs and a 0.9989 exponential decay afterwards. Several examples of predictions made by the trained model on Dataset 2 are shown in Fig. 5 ▸.

**Figure 6:**
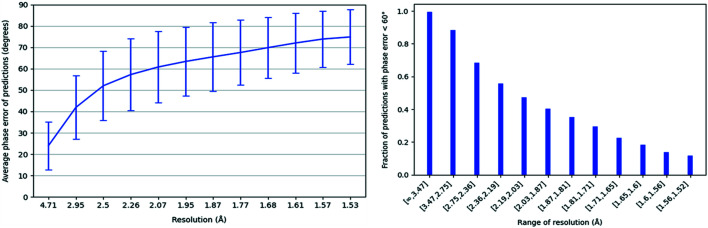
(Left) A plot of the average phase error of validation set predictions on Dataset 2 (dipeptide dataset) against resolution. Note that, unlike for the dialanine datasets, the average phase error rises above 60° before about 2 Å resolution. (Right) The fraction of validation set predictions for which the phase error is <60° at various ranges of resolution, for Dataset 2 predictions.

After performing phase error analysis on the post-training validation set predictions for this dataset, we once again plotted average prediction phase errors against resolution, as shown in Fig. 6 ▸. This plot shows that, in this much more difficult problem, the current model has difficulty reproducing the finer details of the corresponding electron densities. This was expected as we are now working with all of the possible proteinogenic amino acid residues, almost all of which are considerably more complex in structure than alanine. We also generated another plot of the fraction of predictions with phase error <60° at various ranges of resolution, also shown in Fig. 6 ▸. The fraction begins decreasing at lower resolution bins than for Dataset 1a, which shows that phase errors tend to become higher than 60° at lower ranges of resolution than those for the dialanine dataset predictions. However, it also shows that only a very small fraction of the predictions can be considered completely unusable. The model is still able to reproduce the overall shape of the desired electron density the vast majority of the time. A summary of our experimental results, including median Pearson correlation coefficients and median phase errors of validation set predictions after training, is shown in Table 1 ▸.

## 4. Challenges and limitations

We have shown that, at least for simple cases, our CNN-based approach is viable for directly determining structures from Patterson maps. Our eventual goal is to design an algorithmic approach for bypassing the crystallographic phase problem that goes beyond our synthetic cases to more realistic ones. Several challenges will have to be overcome along the way, some of which have some theoretical bases for implementation and some of which need algorithmic development. We also expect that scalability will be a challenge. We may have to look into recent advances in convolutional model architecture or even begin implementing custom convolutional layers. These concerns may also lead us to pursue alternative or novel model architectures in place of our current convolutional U-net setup, which may in turn lead us to approach our problem from a different angle than predicting electron densities from corresponding Patterson maps.

We found that, unlike what was suggested in related work (Hurwitz, 2020 ▸), we do not need to disallow ambiguity in the Patterson vector origin completely when working with our simulated peptide data. However, our most recent datasets still have more empty space around the electron densities than would be considered realistic. Thus, we want to see if our model continues to be able to train as we increase the realism of our datasets.

The current model architecture, along with our current dataset sizes, already requires significant training time overhead with our current computing resources. However, true protein crystal unit cells are still substantially larger than those of the examples in the datasets we have developed. It is also known that convolutional layers scale poorly (order *n* ^3^) with input size (Notchenko *et al.*, 2018 ▸). Thus, we understand that scaling our model to solve realistic protein structures will be a challenge and may require introducing sparsity into our convolutional layers, as in previous work (Notchenko *et al.*, 2018 ▸). Alternatively, or in addition, we may have to begin using dilated convolutions (Yu & Koltun, 2016 ▸) in our convolutional layers, which we previously did not consider to be beneficial for our unique problem.

Our synthetic datasets currently incorporate many simplifying limitations. For example, we trained our model on examples that all have the same cubic unit-cell sizes, but real-world density maps obviously can have different sizes. Although there are methods to include differently sized inputs in CNNs (He *et al.*, 2014 ▸), this is even simpler in our case as we use a U-net architecture that does not end with one or more fully connected layers. Thus, we will not need to change our model architecture to allow for inputs and corresponding outputs of varying rectangular unit-cell sizes. On the other hand, the *PyTorch* framework requires that all examples within a batch must have the same size, so we need to find a workaround for this issue. Furthermore, our current data generation process assumes that all unit-cell angles are exactly 90°. We will also eventually want to create datasets with variable true unit-cell angles (the generated *PyTorch* tensors will still be rectangular) to see if our model can also be robust to this kind of variation, and potentially implement changes to address this.

All of the experiments performed thus far have been in space group *P*1, with no internal symmetry considered. Methods that best include cyclic and dihedral symmetries in CNNs with minimal increases in effective parameters need to be explored. We will adapt our fabricated test cases to include *C*2, *C*4, *D*2 and/or *D*4 symmetries, *e.g.* by modifying the convolutional layers in our neural network and verifying their functionality in solving Patterson maps.

We can also look to introduce additional known data to help our model, which currently only takes entire Patterson maps as input. This is in stark contrast to the approach of *AlphaFold2*, the most important recent related result (Jumper *et al.*, 2021 ▸; Tunyasuvunakool *et al.*, 2021 ▸). In particular, we do not currently make any use of the actual identities and order of the amino acid residues in each example. We could embed sequence data and other information in a 3D tensor and thus provide more than one channel to our model inputs. For example, since convolutional models are known to be robust to the rotational orientation of their inputs (Goodfellow *et al.*, 2016 ▸), we could provide the *n* most common rotamers of the peptides in an example as additional channels.

Another direction we could pursue is to replace some phases of our current convolutional U-net model with new architectures. Although they can be considered to be 3D images, Patterson maps do not actually exhibit any spatial locality, so immediately performing convolution on them may not be the most theoretically sound approach. Thus, we could replace the Encoding phase, or both the Encoding and Learning Features phases, with a 3D vision transformer model (Chen *et al.*, 2021 ▸). Additionally, using simple convolutional layers to produce our model outputs could be the reason why our predicted electron densities tend to be too smooth and lacking in finer details. To address this, we can replace the decoding phase of our model with a diffusion decoder (Ramesh *et al.*, 2022 ▸) or even another transformer.

Finally, for the proof-of-concept work described here, we do not claim that our approach is the best way of actually solving new simple crystal structures. Our resolution is slightly worse than that required for most direct methods, but in fact we could solve a couple of trial examples using *SHELXT* (Sheldrick, 2015 ▸). It seems that molecular replacement could also work. Our longer term goal is to develop a machine learning framework for larger scale, more difficult cases.

## 5. Conclusion

Overall, we have solved Patterson maps from synthetic datasets consisting of short peptides derived from existing PDB entries. This was achieved by the successful training of a convolutional U-net neural network. We have shown the viability of such an approach for solving the structures of simple systems, and have also identified several potential avenues for further research on using neural networks to help solve the crystallographic phase problem.

## 6. Related literature

For further literature related to the supporting information, see Eastman *et al.* (2017 ▸), Read & Schierbeek (1988 ▸), Winn *et al.* (2011 ▸) and Wojdyr (2022 ▸).

## Supplementary Material

Additional details of the process. DOI: 10.1107/S2052252523004293/mf5063sup1.pdf

## Figures and Tables

**Table 1:** The results of reported experiments

Dataset	Training examples	Validation examples	Cell size (Å^3^)	Grid spacing (Å)	Amino acids	Epochs	Median Pearson CC	Median phase error
1a	28 470	3147	20 × 20 × 20	0.5	A	1000	0.93	52°
1b	66 504	7390	10 × 10 × 10	0.5	A	1000	0.98	25°
2	424 096	47 126	12 × 12 × 12	0.6	All 20	200	0.87	64°
