# Supplementary material for: A deep learning solution for crystallographic structure determination
Source: IUCrJ. 2023 Jul 1;10(Pt 4):487–96. doi: 10.1107/S2052252523004293 (PMC10324481; doi:10.1107/S2052252523004293)
Supplement: Supplementary file 1 [file m-10-00487-sup1.pdf]

IUCrJ

Volume 10 (2023)

Supporting information for article:

A deep learning solution for crystallographic structure determination

Tom Pan, Shikai Jin, Mitchell D. Miller, Anastasios Kyrillidis and George N. Phillips, Jr

Supporting information for article:

A Deep Learning Solution for Crystallographic Structure Determination

Tom Pan, Shikai Jin, Mitchell D. Miller, Anastasios Kyrillidis, George N. Phillips, Jr.

1. Details on data generation

The synthetic datasets used in this process were generated as follow, see Figure 1. We first curated a dataset of nearly 24000 representative Protein Data Bank (PDB) entries of proteins solved by X-ray crystallography after 1995, with sequence length $\geq$ 40, refinement resolution ≤ 2.75, and refinement R-Free ≤ 0.28, with clustering at 30% sequence identity. To generate each of our datasets, we began by randomly selecting a subset of 10000-15000 entries from this curated set of PDB entries. For each of these selected entries, we extracted each dipeptide of adjacent amino acid residues and stored the coordinates of these dipeptides in separate .pdb files. For Datasets 1a and 1b, we truncated the side chains of the residues to enforce only dialanine examples. We then randomly selected a small subset of all of these extracted dipeptide coordinates. Using the pdbfixer Python API (Eastman et al., 2017), we removed all dipeptides that contained nonstandard residues or had missing atoms from our set of examples. We then applied several standardized modifications to each of the remaining dipeptide .pdb files: we set all temperature factors to 20.0 and removed all hydrogen atoms leaving only carbon, nitrogen, oxygen, and potentially sulfur.

Afterwards, the coordinates were translated to place the center of mass of the dipeptide in the center of a P1 unit cell of fixed unit cell lengths and with angles set to 90°. Structures that had any atom within 1.5 Å of an atom in an adjacent cell were removed from the dataset. We then calculated structure factors in .mtz format using the gemmi sfcalc program (Wojdyr, 2022) to a resolution of 1.5 Å. From these .mtz files, we created .ccp4 format Patterson and electron density maps using the fft program of the CCP4 program suite (Read & Schierbeek, 1988; Winn et al., 2011), specifying a grid oversampling factor of 3.0. This results in a 0.5-0.6 Å grid spacing in the produced maps. For both the generated Patterson and electron density maps, we converted from .ccp4 format to PyTorch tensor by using the gemmi Python API (Wojdyr, 2022) to

read in the ccp4 maps and copying all the grid points.

We then found the maximum and minimum values in each of the Patterson tensors, as well as the maximum and minimum values in each of the electron density tensors. Finally, we used the maximum and minimum over the values in all tensors to scale our Patterson and electron density maps. In particular, we divided each element in the maps by the difference between the largest maximum and smallest minimum values. This constrained all elements in the tensors of our datasets to be in the range [-1, 1].

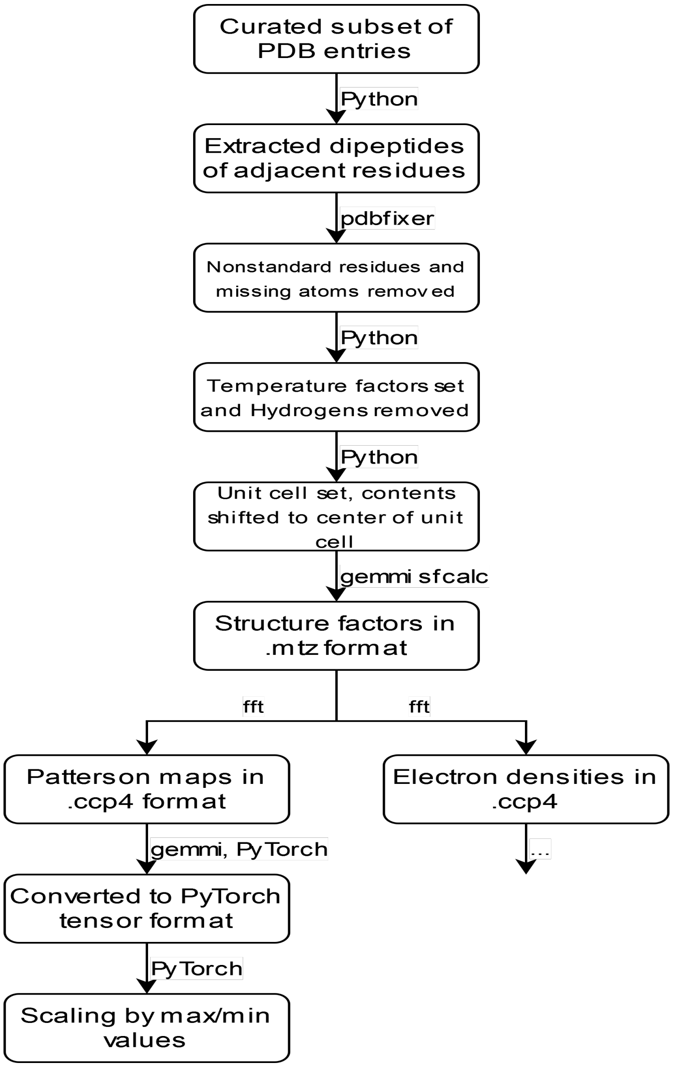

Fig. 1. Visualization of our data generation process

In selecting the dipeptides, effort was taken to ensure diversity by sampling from PDB entities with low sequence similarity to each other. However, both test and training sets are taking random samples from the conformations allowed in rotamer and Ramachandran space. Any similar conformations would be expected to be in a different rotational orientation in the cell by the nature of the selection process. We did not compute all verses all clustering or force the test and training sets to sample distinct conformational regions.

2. Layers of our model and residual blocks

The layers of our model architecture are described in detail in Tables 1 and 2.

Table 1. Layers of the model architecture

Layer	Description	Trainable Parameters
Convolutional	7x7x7 kernels, 23 output channels, 3 padding, 1 stride, 1 dilation, no bias	7889
Batch Normalization		46
ReLU		
Convolutional	7x7x7 kernels, 25 output channels, 3 padding, 1 stride, 1 dilation, no bias	197225
Batch Normalization		50
ReLU		
Max Pooling	2x2x2, 2 stride	
Residual Block	x7	429487
Upsampling	Increase h,w,d dimensions by a factor of 2	
Convolutional	5x5x5 kernels, 23 output channels, 2 padding, 1 stride, 1 dilation, no bias	71875
Batch Normalization		46
ReLU		
Convolutional	5x5x5 kernels, 1 output channel, 3 padding, 1 stride, 1 dilation, with bias	2876
Tanh		

Table 2. Layers of a Residual Block

Layer	Description	Trainable Parameters
Convolutional	7x7x7 kernels, 25 output channels, 3 padding, 1 stride, 1 dilation, no bias	214375
Batch Normalization		50
ReLU		
Convolutional	7x7x7 kernels, 25 output channels, 3 padding, 1 stride, 1 dilation, no bias	214375
Batch Normalization		50
Squeeze and Excitation Block (Hu et al., 2018)	Global reweight of current channels	637
Skip Connection		
ReLU		

3. Algorithmic description of the three phases of our model

The three phases of our model architecture are defined below.

```
Def: EncodingPhase(X); X:Input Patterson map
X = 3d 7x7x7, 23 Channel Convolution(X)
X = ReLU(BatchNorm(X))
X = 3d 7x7x7, 25 Channel Convolution(X)
X = ReLU(BatchNorm(X))
X = 3d 2x2x2 Max Pooling(X)
```

```
Def: LearningFeaturesPhase(X); X:EncodingPhase Output
for i ← 1 to 7 do
    X' = X
    X = 3d 7x7x7, 25 Channel Convolution(X)
    X = ReLU(BatchNorm(X))
    X = 3d 7x7x7, 25 Channel Convolution(X)
    X = BatchNorm(X)
    X = SEBlock(X) (Hu et al., 2018)
    X = X + X'
    X = ReLu(X)
end for
X = 3d Nearest Upsampling(X)
```

```
Def: DecodingPhase(X); X:LearningFeaturesPhase Output
X = 3d 5x5x5, 23 Channel Convolution(X)
X = ReLU(BatchNorm(X))
X = 3d 5x5x5, 1 Channel Convolution(X)
X = Tanh(X)
```

4. Memory and time cost on datasets

Dataset	Training examples	Cell size (Å^3)	GPU Memory Usage (MiB)	Time per epoch (min)
1a	28 470	20x20x20	15409	14.3
1b	66 504	10x10x10	3393	5.1
2	424 096	12x12x12	4151	42.0

IUCr macros version 2.1.15: 2021/03/05

References

Eastman, P., Swails, J., Chodera, J. D., McGibbon, R. T., Zhao, Y., Beauchamp, K. A., Wang, L.-P., Simmonett, A. C., Harrigan, M. P., Stern, C. D. et al. (2017). PLoS computational biology, 13(7), e1005659.

Hu, J., Shen, L. & Sun, G. (2018). In 2018 IEEE/CVF Conference on Computer Vision and Pattern Recognition, pp. 7132–7141. New York, NY, USA: IEEE Press.

Read, R. & Schierbeek, A. (1988). Journal of Applied Crystallography, 21(5), 490–495.

Winn, M. D., Ballard, C. C., Cowtan, K. D., Dodson, E. J., Emsley, P., Evans, P. R., Keegan, R. M., Krissinel, E. B., Leslie, A. G. W., McCoy, A., McNicholas, S. J., Murshudov, G. N., Pannu, N. S., Potterton, E. A., Powell, H. R., Read, R. J., Vagin, A. & Wilson, K. S. (2011). Acta Crystallographica Section D, 67(4), 235–242.

Wojdyr, M. (2022). Journal of Open Source Software, 7(73), 4200.
